# The Incidence of Erosive Esophagitis as a Complication of Pediatric Diabetic Ketoacidosis

**DOI:** 10.1155/2021/6636383

**Published:** 2021-03-05

**Authors:** Sungeeta Agrawal, Serife Uysal, Meghan Fredette, Lisa Swartz Topor, Shara R. Bialo, Michael Herzlinger, Jason Shapiro, Linda K. Snelling, Charlotte M. Boney, Jose Bernardo Quintos

**Affiliations:** ^1^Division of Pediatric Endocrinology, Tufts Children's Hospital, Boston, MA, USA; ^2^Division of Pediatric Endocrinology, Texas Children's Hospital, Houston, TX, USA; ^3^Division of Pediatric Endocrinology and Diabetes, Rhode Island Hospital, Providence, RI, USA; ^4^Division of Pediatric Gastroenterology, Rhode Island Hospital, Providence, RI, USA; ^5^Division of Critical Care, Rhode Island Hospital, Providence, RI, USA; ^6^Department of Pediatrics, Baystate Children's Hospital, Springfield, MA, USA

## Abstract

**Introduction:**

Gastrointestinal (GI) symptoms commonly occur during diabetic ketoacidosis (DKA) and typically resolve with treatment. However, GI complications can persist after DKA resolves. The incidence of upper GI bleeding during DKA in adults has been described, with erosive esophagitis one of the most common lesions. The incidence of GI bleeding or erosive esophagitis in children with DKA has not been previously reported. We performed a retrospective chart review of DKA admissions in children 0 to <18 years with type 1 diabetes mellitus (T1DM) at a pediatric hospital between January 2009 and July 2016. Among 395 episodes of DKA over 7.5 years, erosive esophagitis occurred during two DKA admissions (0.5%) and there were no episodes of GI bleeding. *Case presentations*. Both episodes of erosive esophagitis occurred in adolescent males with known T1DM who presented with severe DKA. Both developed odynophagia after resolution of DKA and were readmitted for DKA recurrence. Upper endoscopy for both patients showed erosive esophagitis. Biopsies were negative for infection, though candida was found during one patient's endoscopy. Both had resolution of their esophagitis symptoms with medication management; neither has had recurrence.

**Conclusion:**

Erosive esophagitis, a rare complication of pediatric DKA, can manifest with odynophagia or substernal chest pain. This complication can lead to DKA recurrence, likely due to increased insulin resistance from inflammation and pain and from reduced oral intake and insulin administration. Patients with odynophagia associated with DKA should be monitored closely to allow timely evaluation and treatment of esophagitis.

## 1. Introduction

The incidence of type 1 diabetes mellitus (T1DM) is rising, and diabetic ketoacidosis (DKA) occurs in approximately 30% of children with new onset T1DM [1]. Common gastrointestinal (GI) symptoms of DKA include abdominal pain, nausea, and vomiting, and these symptoms often resolve with treatment [2]. GI complications that persist after resolution of DKA can also occur, such as pancreatitis and GI bleeding [3]. The incidence of upper GI bleeding in adults with DKA is around 9%, and erosive esophagitis is one of the most common lesions found during endoscopy [4, 5]. The incidence of GI bleeding or acute esophagitis in youth with DKA has not been reported.

The mechanisms of DKA-associated GI bleeding are unknown. Acute hyperglycemia can lead to delayed gastric emptying, resulting in reflux and mucosal damage [6]. Clinical parameters associated with increased risk of GI bleeding during DKA include longer duration of diabetes and diabetes complication such as gastroparesis [5]. Use of proton pump inhibitors and H2 receptor antagonists, suggestive of antecedent symptoms of peptic acid disease, has also been associated with increased risk of GI bleeding during DKA [5]. Laboratory values associated with increased risk of upper GI bleeding during DKA include elevated BUN, creatinine, and glucose [4, 5].

Given the lack of data about GI bleeding and esophagitis during DKA in the pediatric population, we sought to determine the incidence of these GI complications associated with DKA in pediatric patients.

## 2. Case Presentation

### 2.1. Methods

We performed a retrospective cohort study of children 0 to <18 years admitted to Hasbro Children's Hospital for pediatric DKA, between January 1, 2009, and July 31st, 2016, as previously described [7]. DKA was defined in accordance with the International Society of Pediatric and Adolescent Diabetes (ISPAD) consensus guidelines: pH ≤ 7.3 or bicarbonate ≤15 mEq/L (15 mmol/L), with blood glucose ≥200 mg/dl (11.1 mmol/L), and ketosis or ketonuria [8]. Treatment was also in accordance with ISPAD and pediatric guidelines [8, 9]. Information collected included vital signs, anthropometrics, laboratory data, past medical history, physical exam, and complications. Charts for patients with erosive esophagitis were reviewed in detail. REDCap (Research Electronic Data Capture) electronic data capture tools, hosted by Lifespan Corporation, were used to collect and manage study data [10].

### 2.2. Results

We identified 395 episodes of DKA over 7.5 years, with average age 12.7 years (range 0.6–17.9 years). As described previously, 214 of the 395 DKA episodes occurred in males (54%), and 176 episodes occurred in those with new onset diabetes mellitus (NODM) (45%) [7].

### 2.3. Cases

**Figure 1:**
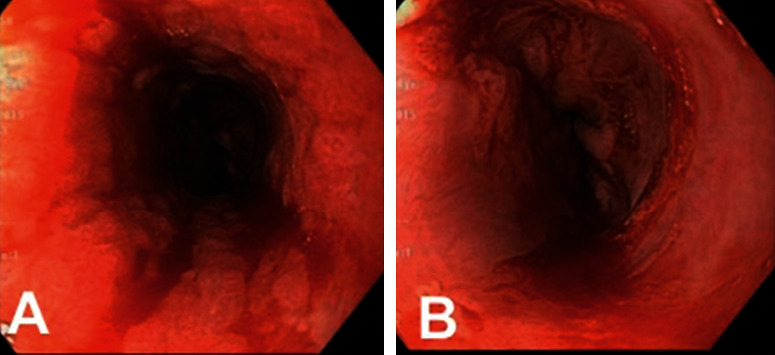
(a) Severe mucosal abnormality with erythema, friability (with spontaneous bleeding), and hemorrhagic appearance in the middle and distal esophagus. (b) Mucosal friability and sloughing in the distal esophagus (both images from Patient B).

Erosive esophagitis occurred in two male patients (0.5%) with known T1DM who presented with severe DKA (Table 1). Neither patient had other complications of diabetes, including microalbuminuria, retinopathy, or neuropathy. Patient A presented with DKA in the setting of inadequate sick day management during illness, and he developed substernal chest pain during hospitalization. He was prescribed ranitidine and carafate and was discharged home. He was readmitted two days later with recurrent DKA and odynophagia. Patient B presented with severe DKA in the setting of a GI illness. Two days after discharge, he was readmitted for management of pancreatitis without DKA. He had abdominal pain at that time, with no episodes of emesis. Pancreatitis resolved, and he developed odynophagia over the next two weeks and was readmitted with recurrent DKA. Upper endoscopy for both patients showed erosive esophagitis.  Figure 1 shows biopsy results for patient B. Biopsies were negative for infection, though brushings from patient B were positive for candida and he was treated with fluconazole. Patient A was empirically treated with fluconazole prior to his endoscopy, and his brushings were negative for candida. Neither had oral candidiasis. Both patients were discharged home on proton pump inhibitors and had resolution of their symptoms; neither has had recurrence during 4 years of follow-up.

## 3. Discussion

Erosive esophagitis is a rare complication of pediatric DKA and occurred in 0.5% of DKA admissions at a pediatric hospital. In both cases, the erosive esophagitis was associated with DKA recurrence. To our knowledge, this is the first study to report the incidence of erosive esophagitis in pediatric DKA.

Abnormal gastric motility may contribute to the development of erosive esophagitis during DKA. Gastroparesis is a known long-term complication of diabetes that occurs due to autonomic neuropathy and is associated with increased risk of GI bleeding during DKA [5]. Gastroparesis affects up to 50% of patients with diabetes in moderate control (both T1 and T2DM) [11]. The T1D Exchange Registry found that 4.8% of adult patients with a diabetes duration of at least 2 years were clinically diagnosed with gastroparesis [11, 12]. The incidence of gastroparesis among children with T1DM has not been reported, and neither of our patients had a diagnosis of gastroparesis. However, acute hyperglycemia can also result in delayed gastric emptying, even without the underlying diagnosis of gastroparesis [6].

Esophageal candidiasis may also contribute to risk of erosive esophagitis during DKA. One of our patients had findings of candida on endoscopy, while the other patient was treated empirically with fluconazole. In adults, diabetes mellitus is a known risk factor for esophageal candidiasis [13, 14] and has been reported in a child with poorly controlled T1DM [15]. Further studies are needed to study the role of candidal infection in esophagitis in DKA.

Both of our patients experienced erosive esophagitis in the setting of DKA recurrence. The esophagitis was likely a contributing factor to the DKA recurrence, due to increased insulin resistance from pain and inflammation [16–18], and reduced insulin administration due to decreased food intake. Therefore, while erosive esophagitis is a rare event, consequences are significant. In patients with DKA and odynophagia, we recommend clinicians consider early GI evaluation, work-up, and management. Prompt treatment can reduce pain, improve oral intake, improve insulin sensitivity, and reduce risk of DKA recurrence.

Interestingly, we did not find any episodes of GI bleeding during DKA, a complication which has been reported in adults with DM. It is possible that erosive esophagitis is a less severe presentation and that treatment prevented subsequent GI hemorrhage or that the manifestation of this type of GI complication differs in pediatric patients compared to adults. In adults with diabetes, those with peptic disease treated with acid suppression are more likely to develop a GI bleed in the setting of DM that those not requiring acid suppression, suggesting that individuals at higher risk for GI bleed may have had antecedent symptoms that led to initiation of acid suppressive therapy [5]. Thus, it is possible that patients with acid reflux symptoms prior to DKA presentation such as heartburn, dysphagia, or odynophagia may be at higher risk for GI bleeding/erosive esophagitis. As erosive esophagitis is a rare event in pediatric DKA, larger studies including multiple diabetes centers are needed to better characterize this complication of DKA.

## 4. Conclusion

While erosive esophagitis is a rare complication of pediatric diabetic ketoacidosis, it is associated with increased morbidity, including DKA recurrence. Patients with odynophagia or substernal chest pain during or after DKA should be evaluated for erosive esophagitis and monitored closely for recurrent ketosis and acidosis.

## Figures and Tables

**Table 1:** Clinical characteristics of patients presenting with esophagitis as a complication of severe diabetes ketoacidosis.

	Patient A	Patient B
Chronologic age (yrs)	16	13
Sex	Male	Male
Diabetes duration (yrs)	7.3	3.5
HbA1c prior to admission (%, (mmol/mol))	12 (108)	7.1 (54)
Average HbA1c over the past year (%, (mmol/mol))	10.2 (88)	7.5 (58)
pH	6.9	6.9
Serum glucose (mg/dl)	217	952
HCO3 (meq/L)	<5	3
Sodium (meq/L)	138	137
Potassium (meq/L)	3	5.8
BUN (mg/dl)	13	54
Creatinine (mg/dl)	0.76	1.9
Upper GI endoscopy	Moderate to severe esophagitis in entire esophagus	Severe esophagitis in entire esophagus

## Abbreviations

T1DM: Type 1 diabetes mellitus
DKA: Diabetic ketoacidosis
GI: Gastrointestinal
ISPAD: International society of pediatric and adolescent diabetes
REDCap: Research electronic data capture
NODM: New onset diabetes mellitus.
